# Socioeconomic Inequality in One-Year Mortality of Elderly People with Hip Fracture in Taiwan

**DOI:** 10.3390/ijerph15020352

**Published:** 2018-02-16

**Authors:** I-Lin Hsu, Chia-Ming Chang, Deng-Chi Yang, Ya-Hui Chang, Chia-Chun Li, Susan C. Hu, Chung-Yi Li

**Affiliations:** 1 Department of Public Health, College of Medicine, National Cheng Kung University, Tainan 701, Taiwan; yilinhsu@gmail.com (I.-L.H.); yhccat@gmail.com (J.-H.C.); ghtjulia@gmail.com (C.-C.L.); shuhu@mail.ncku.edu.tw (S.C.H.); 2 Department of Emergency Medicine, National Cheng Kung University Hospital, College of Medicine, National Cheng Kung University, Tainan 701, Taiwan; 3 Division of Geriatrics and Gerontology, Department of Internal Medicine, National Cheng Kung University Hospital, College of Medicine, National Cheng Kung University, Tainan 701, Taiwan; charming.tw@hotmail.com; 4 Institute of Gerontology, College of Medicine, National Cheng Kung University, Tainan 701, Taiwan; 5 Department of Public Health, College of Public Health, China Medical University, Taichung 404, Taiwan

**Keywords:** hip fracture, mortality, socioeconomic status, urbanization, health inequality

## Abstract

Hip fracture commonly results in considerable consequences in terms of disability, mortality, long-term institutional care and cost. Taiwan launched its universal health insurance coverage in 1995, which largely removes financial barriers to health care. This study aims to investigate whether socioeconomic inequality in one-year mortality exists among Taiwanese elderly people. This population-based cohort study included 193,158 elderly patients (≥65 years) admitted for hip fracture between 2000 and 2012. With over a one-year follow-up, 10.52% of the participants died from all causes. The mortality rate was low in the northern part of Taiwan and in urban and high-family-income areas. Multiple Poisson regression models further suggested that the level of >Q1–Q3 and >Q3–Max showed significantly reduced odds ratio of one-year mortality at 0.90 (95% confidence interval (CI), 0.87–0.93) and 0.77 (95% CI, 0.74–0.81), respectively, compared with that of the lowest family income level (i.e., Min.–Q1). Despite a monotonic decline in overall one-year mortality during the study period, socioeconomic inequality in one-year mortality rate remained evident. The annual percentage change in one-year mortality was higher (−2.86) in elderly people from families with high income (>Q3–Max.) than that for elderly patients from family with low income (Min.–Q1, −1.94). Accessibility, rather than affordability, to health care for hip fracture is probably responsible for the observed socioeconomic inequality.

## 1. Introduction

Hip fracture commonly results in considerable consequences in terms of disability, mortality, long-term institutional care, and cost [1,2]. Although the majority of patients who undergo surgical treatment may survive, the proportion of those not completely recuperating to their previous levels of independence remains high [3]. Given the remarkable increase in the elderly population in many developed nations, hip fractures may exponentially increase in frequency over the next several decades as a result of increased life expectancy and population growth. Additionally, the economic influence of hip fractures may be large; this influence includes mortality and direct cost of medical cares and certain indirect economic burden, such as reduced quality of life, loss of independence, and inability to work [4].

In addition to a general consensus in the literature that mortality due to hip fracture is associated with age, sex, comorbidity, functional status, dementia, arrhythmia, and congestive heart failure, certain system factors affecting the mortality of patients with hip fracture have also been proposed; these factors include hospitalization delay, July admission, surgical delay, anesthetic type, intensive care admission, hospital volume, surgeon volume, nursing volume, and length of stay [5,6,7,8]. Some of these systematic factors, such as hospital and surgical delays, are associated with patient’s socioeconomic status, which is a well-established factor influencing the health outcomes of patients, including those with hip fracture [9,10,11,12]. Sufficient evidence is available to support race- and insurance-based disparities in delivery of care for patients with hip fracture; some of these disparities persist after adjusting for social deprivation [11,13]. Patient’s socioeconomic status is an etiological factor in the development of hip osteoarthritis and a determinant of access to and outcome of operations; most disadvantaged patients are less likely to undergo total hip replacement, achieve timely access to intervention, or obtain positive outcomes [14,15,16,17].

A recent study conducted in Taiwan showed that the total number of hip fracture cases increased steadily from 12,479 in 1996 to 19,841 in 2010; the trend shows an initial increase and a subsequent decrease in hip fracture rates (from 457.9 to 390.0 fractures per 100,000 people per year) [18]. Given that Taiwan introduced its universal health insurance program in 1995 to cover all residents, this country is a good setting for further examination of the role of socioeconomic status and clinical outcomes among patients with hip fracture. The National Health Insurance (NHI) program aims to ensure the accessibility to health care at a reasonable cost [19]; this program extends the existing insurance coverage from 57% of the population (mostly employed individuals) to the whole population; consequently, several segments of the population, including the children, elderly, and nonworking adults, considerably benefited from the NHI program. The lack of insurance is associated with increased morbidity and mortality [20,21]; hence, the implementation of the NHI program may improve the outcomes of various diseases and injuries, including hip fracture.

Apart from financial barriers, there are some other factors that may limit timing accessibility to surgery for hip fracture in older patients. A US study indicated that the primary reasons for delaying surgery more than 24 h after hospital arrival were waiting for routine medical clearance and unavailability of the operating suite or surgeon. In addition, stabilization of associated medical conditions could also result in a longer period of delay [22]. Because both affordability and accessibility to health care are associated with socioeconomic status, and the universal coverage of health insurance in Taiwan has greatly removed the financial barriers to health care. In the present study, we aimed to examine the secular trend in one-year mortality during the implementation of the NHI program and assess whether the risk of one-year mortality still shows a socioeconomic disparity in Taiwan. 

## 2. Materials and Methods

### 2.1. Data Source

This study was approved by the Research Ethics Committee of the National Cheng Kung University (approval number 103-010). The data analyzed in this study were inpatient claims retrieved from Taiwan’s National Health Insurance Research Data (NHIRD), as provided by the National Health Insurance Administration (NHIA), Ministry of Health and Welfare, Taiwan. Approximately 92.3% of Taiwan residents were enrolled in the NHI program by 1996, and the coverage has increased to 99% since 2004 [23]. The medical claims of NHIRD included each patient’s demographic characteristics, residential city/township, codes of disease diagnosis and procedure, prescription records, and medical expenditures. The personal identification numbers of all beneficiaries were encrypted to ensure privacy. To ensure the accuracy of claim files, the NHIA performs quarterly expert reviews on a random sample for every 50–100 ambulatory and inpatient claims [23]. Access to research data was reviewed and approved by the Review Committee of the National Health Research Institutes.

### 2.2. Study Cohort, Follow up, and Endpoint 

Based on the unselected NHIRD, a total of 337,574 admissions for hip fracture (International Classification of Disease Ninth version Clinical Modification (ICD-9-CM) codes: 820.xx) by 291,150 patients aged ≥65 years were noted in Taiwan between 1998 and 2012. We retained the first-time admission for each patient and included only admissions that occurred between 2000 and 2012 (*n* = 245,059 admissions/patients). After excluding those patients aged 64 or less, the remaining 193,158 elderly patients were included as the study cohort. The study participants were considered patients with newly diagnosed hip fracture between 2000 and 2012. All study participants were followed up for 1 year, which started from discharge date of the first-time hospitalization between 2000 and 2012. All-cause mortality was used as the study endpoint. 

### 2.3. Measures of Socioeconomic Status and Covariates

The socioeconomic status of individual study participant was indicated by the urbanization level and median family annual income of the city/township where the participant lived. The urbanization level of each of the 368 cities/townships in Taiwan during enrollment was determined by the method of Liu et al. [24]; these authors classified all cities and townships of Taiwan into seven ordered levels of urbanization according to various indicators, including population density, proportion of residents with college or higher education, percentage of elderly (>65 years) people, proportion of the agricultural workforce, and number of physicians per 10^5^ people. We also used the median family annual income of each city/township to indicate the neighborhood socioeconomic status of the study participant. Information on the median family annual income of individual city/township during the study period was obtained from the Government Open Data, as supervised by Taiwan National Development Council (http://data.gov.tw/node/17983). 

Two categories of risk factors associated with the risk of one-year mortality in patients with hip fracture were considered as covariates. Patient-level characteristics included gender, age, and selected comorbidities, including dementia (ICD-9-CM codes: 331.0x or 290.xx), stroke (ICD-9-CM codes: 430.xx-438.xx), chronic obstructive pulmonary disease (ICD-9-CM codes: 490.xx-492.xx, 496.xx), and heart failure (102.01, 402.11, 402.91, 404.01, 404.03, 404.11, 404.13, 404.91, 404.93, 428.xx). These comorbidities are involved in the proposed biological mechanisms of the association between hip fracture and death [8,25,26]. Information about the selected comorbidities was retrieved from each study subject’s inpatient and outpatient claims within the three-year period prior to the date of discharge from the index admission for hip-fracture.

Hospital-level characteristics considered in this study included hospital accreditation level and length of hospital stay for the first-time hip fracture. All hospitals in Taiwan are accredited into medical centers, regional hospitals, and local hospitals, according to service volume and teaching status. We determined a study participant’s the socioeconomic status of residential city/township and hospital-level characteristics based on the information of the year when hip fracture admission occurred.

### 2.4. Statistical Analysis

We first calculated the overall and stratified one-year mortality rates according to socioeconomic indicators or other covariates. Pearson’s χ^2^ test was used to compare characteristics between the study participants who died in one year after surgery and those who survived. We subsequently used unconditional logistic regression models to estimate the crude and covariate-adjusted odds ratios (ORs) and their 95% confidence intervals (CIs) of one-year mortality in association with the socioeconomic indicators.

To account for city/township clustering, the association of urbanization level with one-year mortality was analyzed using logistic regression model with generalized estimating equation methods [27], which specify an exchangeable structure of a working correlation matrix to construct regression models. The binary outcomes were regressed with a *logit* link function. Both univariate and multivariate analyses were performed to obtain the crude and adjusted ORs. We used sequential approach in multivariate regression models to assess the independent effect of socioeconomic status measures on one-year mortality, in which patient-level characteristics were first included in Model 1, then both patient-level and hospital-level characteristics were simultaneously included in Model 2. This way of doing may help evaluate the potential confounding by patient-level and hospital-level characteristics, respectively. The multicollinearity among socioeconomic measures and covariates was examined, and no large estimated slope coefficients or estimated standard error of means were observed [28]. To assess the potential effect-modification by age on the relationship between socioeconomic status and one-year mortality, we performed stratified analysis to look into age-specific effect of median family annual income on one-year mortality.

To assess the socioeconomic inequality in one-year mortality caused by hip fracture across the study period, we presented the secular trends for one-year mortality rate in relation to overall and median family annual income level. The significance of linear trend was further evaluated with jointpoint regression model, which also calculated the annual percentage change (APC) in one-year mortality across time [29]. The joinpoint regression model is preferable when analyzing the trend of mortality or incidence for several years as it enables the identification points in the trend where the significant changes occur. Additionally, getting the smooth mortality (or incidence) curves including the capability of detecting change points is useful to actuaries and policy makers [30]. The APC was used to characterize the behavior of the cancer trends. The estimated APC is the percentage change (increase or decrease) in the estimated mortality rates per year in the time trend. More specifically, it estimates the rate of change of mortality rate from *t*th year to *(t + 1)*th year. This measure helps us to compare the mortality trend among the different subpopulations across time.

Statistical analyses were performed using SAS (Version 9.4; SAS Institute, Cary, NC, USA) and Joinpoint Trend Analysis Software (Ver 4.5.0.1; National Cancer Institute, Rockville, MD, USA). A *p* value of <0.05 was considered statistically significant.

## 3. Results

The overall one-year mortality rate of hip fracture was 10.52% among patients admitted during 2000–2012 in Taiwan. Table 1 shows one-year mortality rates according to residential city/township and patient- and hospital-level characteristics. Result showed geographic disparity, where patients living in the Northern and Eastern Taiwan experienced the lowest (9.75%) and highest (12.11%) mortality rates, respectively. The socioeconomic inequality was evident in that patients from urban areas showed a mortality rate of 9.91%, which was lower than those in satellite (10.23%) and rural areas (11.27%). A similar socioeconomic gradient relationship was noted when city/township median family income was used alternatively as the socioeconomic indicator. 

Patients living in the North, Central, and South were all less likely to encounter one-year mortality compared with those from the East, with a crude OR of 0.78 (95% CI, 0.73–0.84), 0.92 (95% CI, 0.85–0.99), and 0.87, (95% CI, 0.81–0.94), respectively. With consideration of patient- and hospital-level characteristics, only patients from the North still showed a significantly reduced OR (0.87, 95% CI, 0.80–0.947). Both urban (0.87, 95% CI, 0.84–0.90) and satellite (0.90, 95% CI, 0.87–0.93) areas were associated with significantly lower crude ORs of one-year mortality rate than those with rural areas. However, such significantly reduced risk disappeared, and covariates were adjusted. Median family income was significantly associated with both crude and covariate adjusted ORs of one-year mortality. The level of >Q1–Q3 and >Q3–Max. showed significantly reduced crude OR at 0.90 (95% CI, 0.87–0.93) and 0.77 (95% CI, 0.74–0.81), respectively, compared with that of the lowest median family income level (i.e., Min.–Q1). Such inverse relationship between median family income level and one-year mortality remained unchanged after adjustment for both patient- and hospital-level characteristics (Table 2). Table 2 also shows an OR of one-year mortality in association with both patient- and hospital-level characteristics. Male patients, old patients, and patients with selected comorbidities, including dementia, stroke, COPD, and heart failure, were all associated with significantly increased OR of one-year mortality. Significantly higher ORs were also observed in patients treated at medical institutes than those of patients treated at medical centers and those with long hospital stay. Given a significant interaction of age and socioeconomic status, Table 3 further shows the effect of median family annual income on one-year mortality according to a patient’s age. Similar to the findings based on the whole study sample, we noted an inverse relationship between median family annual income and risk of one-year mortality in patients of all age groups, especially in those aged 75 years and older. To look into detailed gradient relationship median family annual income on one-year mortality, we re-categorized the median family annual income into deciles. Supplementary Figure S1 shows an apparent tendency whereby one-year mortality from hip fracture decreased gradually with increasing deciles.

**Figure 1:**
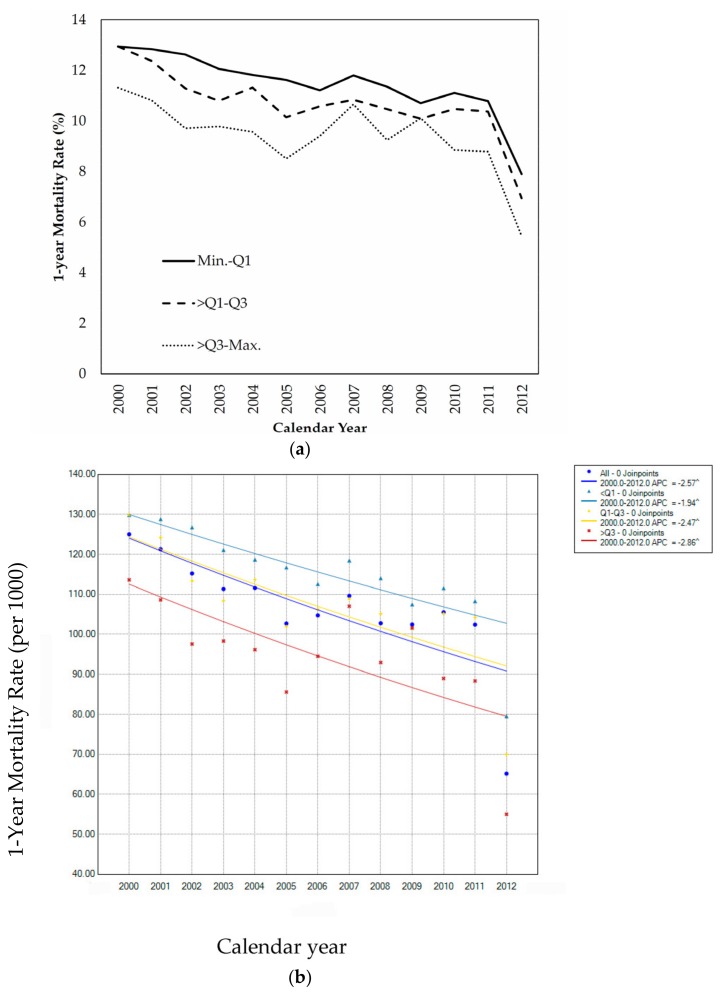
(**a**) Secular trend in socioeconomic disparity of one-year mortality rate from 2000 to 2012. (**b**) Jointpoint regression models estimating the annual percent change across the study period.

Despite a monotonic decline in overall hip fracture one-year mortality over the study period, the socioeconomic inequality of mortality remained evident. Elderly people living in areas with high median family income constantly experienced lower mortality rate than those from areas with low median family income (Figure 1a). Jointpoint regression analysis further indicated a gradient relationship between median family annual income and APC of one-year mortality rate, where the elderly people from areas with high median family income showed the largest reduction in APC (i.e., −2.86) (Figure 1b).

## 4. Discussion

Our study detected a persistent decline in overall one-year mortality rate of patients with hip fracture during the period of universal health coverage in Taiwan, that is, from 2000 to 2012. However, such decline showed socioeconomic inequality, where elderly people from areas with high family income presented the largest reduction in mortality. Furthermore, after years of universal health insurance coverage, the socioeconomic inequality in mortality due to hip fracture remained. The one-year mortality for the family income level of >Q1–Q3 and >Q3–Max. was significantly lower by 10% and 23%, respectively, than that for the lowest family income level (i.e., Min.–Q1).

Socioeconomic inequalities in healthcare quality have been extensively documented [31]. In particular, disadvantaged people show high risk of mortality for a wide range of procedures [32]. Many studies identified specific preoperative indicators for long-term outcome of hip fracture based on background demographic, social, health variables, living conditions, and functional variables (such as premorbid state and daily living activities) [26,33,34]. With considerably few exceptions [35,36], most studies reported a high mortality due to hip fracture in patients with low socioeconomic levels [11,12]. Although our study took into account demographic and health variables such as co-morbidity in the analysis, certain variables including living conditions and physical functioning were not available from claim data, which limits interpretation of our study findings. Among adults undergoing hip fracture surgery, increased waiting time is associated with a high risk of 30-day mortality and other complications. A waiting time of 24 h may represent a threshold defining high risk [37]. Petrelli et al. found that low socioeconomic levels are associated with high risk of waiting >2 days (adjusted relative risk: 1.14), high odds for 90-day (adjusted OR: 1.18), and one-year mortality (adjusted OR: 1.27) [17]. Barone et al. also noted that low socioeconomic level is significantly associated with low chance of early intervention (adjusted relative risk: 0.32) and high risk of mortality (adjusted relative risk: 1.51). Moreover, Dy et al. indicated that Medicaid patients are at significantly increased risk for delayed surgery (OR: 1.17) compared with that of Medicare patients; on the contrary, privately insured patients are at decreased risk for delayed surgery (OR: 0.77), complications (OR: 0.80), and one-year mortality (hazard ratio: 0.80) [11]. An earlier Taiwanese study that included 409 patients (mean age, 72.5 years) with hip fracture from an urban hospital reported that 86% of surgery was performed in 24 h [38]. This number is much higher than that (33.6%) reported in a Canadian study that included 42,230 adults (mean age, 80.8 years) undergoing hip fracture surgery in Ontario between 1 April 2009, and 31 March 2014 [37]. A recent study from Hong Kong identified 43,830 patients with age ranging from 65 to 112 years (mean, 82 years) who presented to any public hospital between January 2000 and December 2011 with hip fracture that was treated surgically. It reported that 48% of surgeries were performed in 48 h [38]. Although limited in scope, data from the above-mentioned hospital-based Taiwanese study tended to suggest a low prevalence of delayed surgery among patients with hip fracture in Taiwan [39]. Because there have been no studies that compared differences in surgical delay among patients with different socioeconomic background, we were unable to conclude whether a long waiting time for surgery can contribute to the observed socioeconomic inequality noted in our study. In addition to preoperative indicators, certain postoperative events are also associated with risk of mortality. A local Taiwanese study noted that the six-year cumulative incidence of a second hip fracture is high in both female (8.0%) and male (6.2%) patients. A significantly higher one-year mortality rate is observed after a second hip fracture (18.8%) than that with the first hip fracture (14.1%). Additionally, men show higher one- and five-year mortality rates after second hip fractures (12.1% and 41.2%, respectively) than those of women (17.4% and 47.3%, respectively) [40]. In a recent Danish population-based cohort study, Kristensen et al. [12] reported that patients with both high education and high income are associated with a reduced risk of acute readmission after hip fracture (14.5% vs. 16.9%). A high risk of readmission for hip fracture may have contributed to the inverse relationship between socioeconomic indicator and hip fracture mortality observed in our study. Our study is the first to investigate the socioeconomic inequality of one-year mortality due to hip fracture in the elderly population of Taiwan. This study is particularly notable given a universal health insurance coverage that has been implemented in Taiwan for many years. Our findings are in agreement with those of Barone et al. who reported that individuals living in disadvantaged census tracts show poor prognoses; these patients are also less likely to be treated according to clinical guidelines despite universal healthcare coverage than those of affluent people [41]. Thus, our study presents important healthcare and policy implications in terms of improving accessibility to health care for those socioeconomically disadvantageous elders with hip fracture. Despite the importance of our findings, our study also suffered from inadequate decomposition of factors that may affect the observed socioeconomic inequality. Both preoperative and post-operated factors can contribute to the socioeconomic inequality in mortality caused by hip fracture.

## 5. Conclusions

Our data demonstrated a socioeconomic inequality in one-year mortality among Taiwanese elderly patients admitted for hip fracture. We also observed a monotonically decreasing one-year mortality rate in Taiwanese elderly population in the era of universal health care program, which considerably removes financial barriers to health care. Despite that fact, like many other countries with different healthcare systems, the socioeconomic inequality in one-year mortality from hip fracture still existed in Taiwan and deserves attention. Future local studies should be carried out to further investigate the determinants, especially those non-financial ones, contributing to such socioeconomic inequality, which will consequently help develop targeted interventions to mitigate the adverse effects of low socioeconomic status on mortality caused by hip fracture among elderly population.

## Figures and Tables

**Table 1:** Characteristics of the study patients and risk of one-year mortality.

Characteristics	No. of Patients ^a^	One-Year Mortality		*p*-Value
	*n* (%)	Number	%	
*Characteristics of residential city/township*				
Geographic area				
North	77,375 (40.1)	7547	9.75	<0.0001
Central	51,095 (26.5)	5743	11.24	
South	56,247 (29.1)	6038	10.73	
East	7483 (3.9)	906	12.11	
Urbanization status				
Urban area	65,254 (33.8)	6469	9.91	<0.0001
Satellite area	51,482 (26.7)	5268	10.23	
Rural area	75,135 (38.9)	8468	11.27	
City/township median family annual income ^b^				
Min.–Q1	42,374 (21.9)	4974	11.74	<0.0001
>Q1–Q3	87,410 (45.3)	9319	10.66	
>Q3–Max.	43,459 (22.5)	4043	9.30	
Patient-level characteristics				
Gender				
Female	118,370 (61.3)	10,134	8.56	<0.0001
Male	74,748 (38.7)	10,192	13.64	
Age, years				
65–74	48,321 (25.0)	3494	7.23	<0.0001
75–84	93,310 (48.3)	9241	9.90	
85+	51,527 (26.7)	7593	14.74	
Comorbidity				
Dementia	7515 (3.9)	999	13.29	<0.0001
Stroke	23,526 (12.2)	2809	11.94	<0.0001
COPD ^c^	16,223 (8.4)	2966	18.28	<0.0001
Heart failure	13,060 (6.8)	2773	21.23	<0.0001
Hospital-level characteristics				
Hospital accreditation level				
Medical center	54,405 (28.2)	5305	9.75	<0.0001
Regional hospital	85,769 (44.4)	8981	10.47	
Local hospital	52,978 (27.4)	6042	11.40	
Length of hospital stay ^d^				
Min–Q1	51,974 (26.9)	5269	10.14	<0.0001
Q1–Median	45,679 (23.7)	3385	7.41	
Median–Q3	50,959 (26.4)	4306	8.45	
Q3–Max	44,546 (23.1)	7368	16.54	
Mean ± SD	10.34 ± 16.44			
Total	193,158 (100.0)	20,328	10.52	

^a^ Inconsistency between the total population and population summed for individual variables was due to missing information. ^b^ Min., Q1, Q3, and Max. for family annual income was 10,000, 16,433, 20167, and 31,066 USD, respectively. ^c^ COPD, chronic obstructive pulmonary disease. ^d^ Min., Q1, Median, Q3, and Max. for length of hospital stay was 1, 6, 8, 11, and 240 days, respectively.

**Table 2:** Odds ratios of one-year mortality in relation to patient- and hospital-level characteristics and residential city/township characteristics.

Characteristics		Odds Ratio	
	Crude Estimate (95% CI)	Adjusted Estimate (95% CI)	
		Model 1 ^a^	Model 2 ^b^
*Characteristics of residential city/township*			
Geographic area			
North	0.78 (0.73–0.84)	0.86 (0.79–0.94)	0.87 *** (0.80–0.94)
Central	0.92 (0.85–0.99)	0.96 (0.89–1.04)	0.97 (0.90–1.05)
South	0.87 (0.81–0.94)	0.98 (0.91–1.07)	0.99 (0.91–1.07)
East (Ref.)	1.00	1.00	1.00
Urbanization status			
Urban area	0.87 (0.84–0.90)	1.04 (0.99–1.09)	1.05 (1.00–1.10)
Satellite area	0.90 (0.87–0.93)	0.98 (0.94–1.03)	1.00 (0.96–1.05)
Rural area (Ref.)	1.00	1.00	1.00
City/Township median family annual income			
Min.–Q1 (Ref.)	1.00	1.00	1.00
>Q1–Q3	0.90 (0.87–0.93)	0.91 (0.87–0.94)	0.91 *** (0.87–0.95)
>Q3–Max.	0.77 (0.74–0.81)	0.80 (0.75–0.85)	0.80 *** (0.75–0.85)
*Patient-level characteristics*			
Gender			
Female (Ref.)	1.00	1.00	1.00
Male	1.69 (1.64–1.74)	1.67 (1.62–1.72)	1.67 *** (1.61–1.72)
Age, years			
65–74 (Ref.)	1.00	1.00	1.00
75–84	1.41 (1.35–1.47)	1.36 (1.31–1.42)	1.37 *** (1.31–1.43)
≥85	2.22 (2.13–2.31)	2.18 (2.08–2.28)	2.18 *** (2.08–2.28)
Comorbidity			
Dementia	1.32 (1.23–1.41)	1.15 (1.06–1.23)	1.12 ** (1.04–1.20)
Stroke	1.18 (1.13–1.23)	1.13 (1.08–1.18)	1.10 *** (1.05–1.15)
COPD	2.06 (1.97–2.15)	1.49 (1.42–1.57)	1.41 *** (1.34–1.48)
Heart failure	2.50 (2.39–2.61)	2.25 (2.15–2.37)	2.11 *** (2.01–2.21)
*Hospital-level characteristics*			
Hospital accreditation level			
Medical center	1.00		1.00
Regional hospital	1.08 (1.04–1.12)		1.05 ** (1.01–1.10)
Local hospital	1.19 (1.15–1.24)		1.14 *** (1.09–1.19)
Length of hospital stay ^c^			
Min–Q1	1.00		1.00
Q1–Median	0.71 (0.68–0.74)		0.71 *** (0.67–0.74)
Median–Q3	0.82 (0.78–0.85)		0.79 *** (0.76–0.83)
Q3–Max	1.76 (1.69–1.82)		1.59 *** (1.53–1.66)

^a^ Min., Q1, Q3, and Max. for median family annual income was 10,000, 16,433, 20167, and 31,066 USD, respectively. ^b^ COPD, chronic obstructive pulmonary disease. ^c^ Min., Q1, Median, Q3, and Max. for length of hospital stay was 1, 6, 8, 11, and 240 days, respectively. *** *p*-value < 0.001; ** *p*-value < 0.01

**Table 3:** Odds ratios of one-year mortality in relation to median family annual income levels according to a patient’s age.

Age Groups, Years	City/Township Median Family Annual Income	Adjusted Odds Ratios (95%CI)
65–74	Min.–Q1 (Ref.)	1.00
	>Q1–Q3	0.89 (0.80–0.98)
	>Q3–Max.	0.84 (0.72–0.97)
75–84	Min.–Q1 (Ref.)	1.00
	>Q1–Q3	0.92 (0.87–0.98)
	>Q3–Max.	0.79 (0.72–0.86)
≥85	Min.–Q1 (Ref.)	1.00
	>Q1–Q3	0.91 (0.85–0.97)
	>Q3–Max.	0.80 (0.73–0.89)

## Supplementary Materials

The following are available online at http://www.mdpi.com/1660-4601/15/2/352/s1, Figure S1: Odds ratios of one-year mortality in relation to deciles of median family annual income.
