# Supplementary material for: Socioeconomic Inequality in One-Year Mortality of Elderly People with Hip Fracture in Taiwan
Source: Int J Environ Res Public Health. 2018 Feb 16;15(2):352. doi: 10.3390/ijerph15020352 (PMC5858421; doi:10.3390/ijerph15020352)
Supplement: Supplementary file 1 [file ijerph-15-00352-s001.docx]

**Supplemental Figure S1.** Odds ratios of one-year mortality in relation to deciles of median family annual income.

| 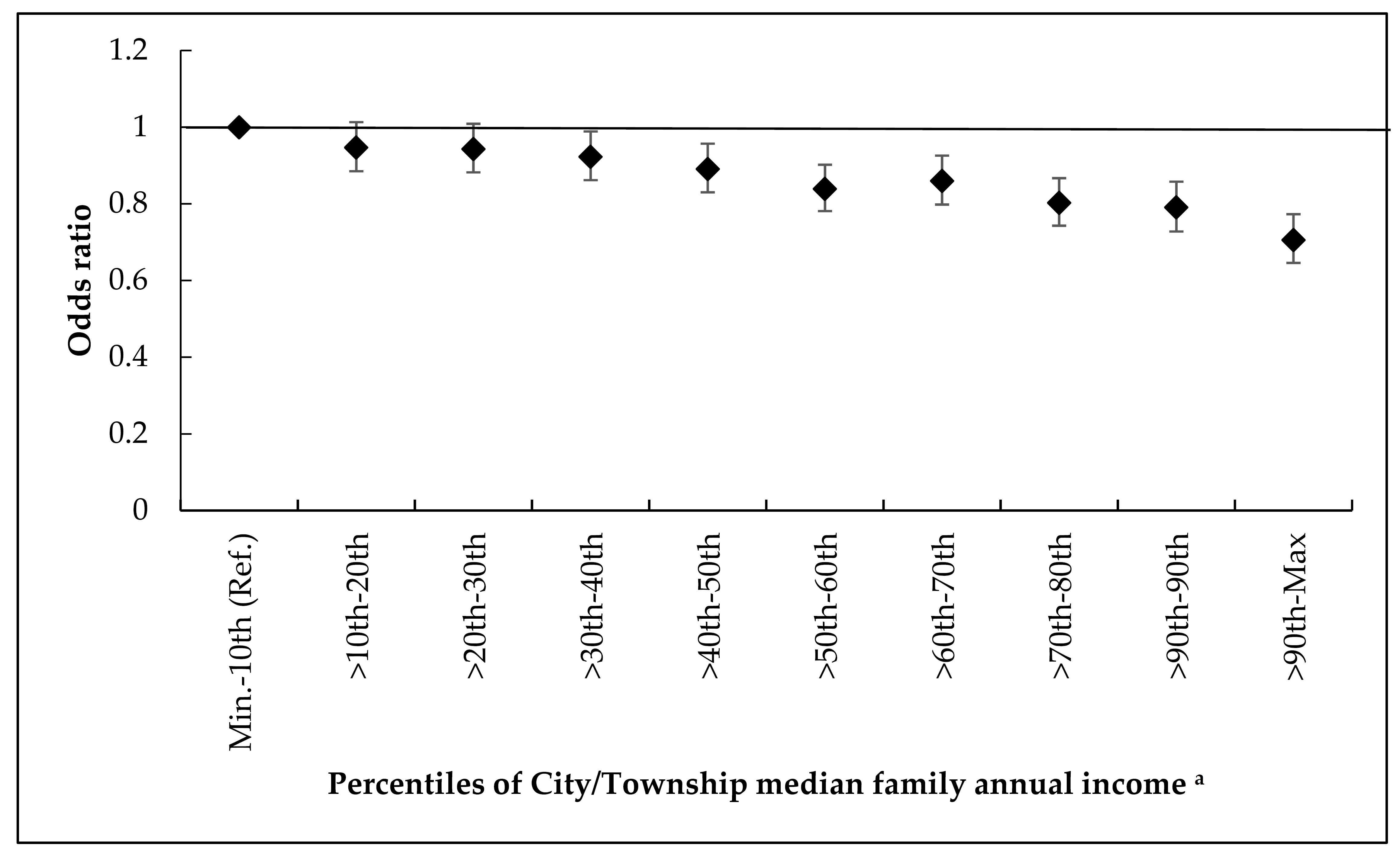  ^a^ The 10^th^, 20^th^, 30^th^, 40^th^, 50^th^, 60^th^, 70^th^, 80^th^, and 90^th^ percentile was 15,400, 16,133, 16,733, 17,433, 18,100, 18,766, 19,700, 20,866, and 23,200 US dollars, respectively. |
| --- |
